# Parkinson's Disease in the Era of a Novel Respiratory Virus Pandemic

**DOI:** 10.3389/fneur.2020.00995

**Published:** 2020-09-11

**Authors:** Matilde Otero-Losada, Tamara Kobiec, Lucas Udovin, Guenson Chevalier, Cecilia Quarracino, Camila Menéndez Maissonave, Sofia Bordet, Francisco Capani, Santiago Perez-Lloret

**Affiliations:** ^1^Biomedical Research Center, Interamerican Open University (CAECIHS-UAI), National Research Council (CONICET), Buenos Aires, Argentina; ^2^Centro de Investigaciones en Psicología y Psicopedagogía (CIPP), Facultad de Psicología y Psicopedagogía, Pontificia Universidad Católica Argentina (UCA), Buenos Aires, Argentina; ^3^Instituto Universitario de Ciencias de la Salud, Fundación H.A Barceló, Buenos Aires, Argentina; ^4^Department of Biology, John F. Kennedy University, Buenos Aires, Argentina; ^5^Facultad de Medicina, Universidad Autónoma de Chile, Santiago, Chile; ^6^Faculty of Medical Sciences, Pontificia Universidad Católica Argentina, Buenos Aires, Argentina

**Keywords:** Parkinson's disease, COVID 19, viral infection, influenaza virus, neurological impact, SARS-CoV-2, coronavirus, neurological diseases

Humankind has gone through major airborne virus pandemics in the modern era. Coronavirus outbreaks have been registered in 2003 [severe acute respiratory syndrome (SARS)], 2009 [Middle East respiratory syndrome (MERS)], and 2019/2020 ongoing [CoV disease (COVID-19)]. Influenza outbreaks were documented in 1918 (post-World War I Spanish flu, H1N1 virus), 1957–1958 (Asian flu, H2N2 virus), 1968 (the Hong Kong flu, H3N2 virus), and 2009 (the swine flu, H1N1 virus). These viruses can only affect humans after mutating in their usual animal hosts, presenting as a zoonotic disease in the beginning. Unknown to the human immune system, they spread swiftly, resulting in outbreaks. Fatality rates vary from >30% for MERS, ~10% for the 1918 Spanish flu, 10% for SARS to <1% for the 2009 MERS. The number of infected people was 700 or 500 million with 2009's or 1918's H1N1 virus, respectively, 8,000 with SARS-CoV-1, and 2,500 with MERS-CoV. As of June 29th, according to the World Health Organization's daily situation report no. 161, SARS-CoV-2 has infected 10 million people, with a 4.98% case fatality rate (https://www.who.int/emergencies/diseases/novel-coronavirus-2019/situation-reports).

Coronavirus (CoV) and influenza virus (IV) are neurotropic (1, 2). Experimental studies using transgenic mice showed that SARS- and MERS-CoV intranasal administration was followed by the invasion of the olfactory neuroepithelium and, ultimately, of the brain (2). Coronavirus could also access the central nervous system by transsynaptic transfer, starting at peripheral nerve endings (2). At later stages, invasion via the bloodstream likely involves angiotensin-converting enzyme 2 (ACE2) receptors (1). A schematic representation can be found in Figure 1. Noteworthy, the brainstem shows the highest level of viral particles. Impairment of the cardiorespiratory nuclei, the nucleus ambiguous and the nucleus of the solitary tract, in particular, may contribute to respiratory distress (2).

**Figure 1:**
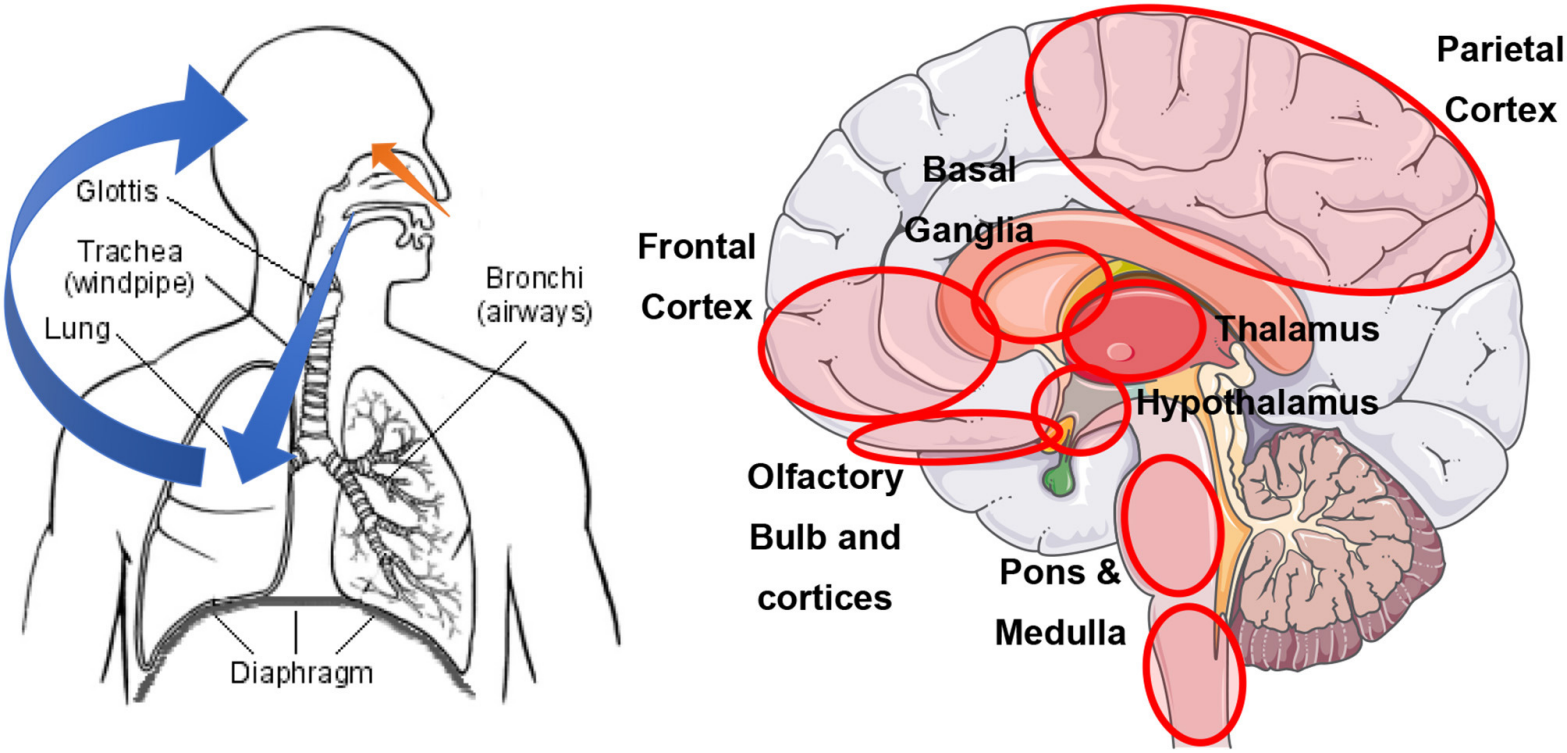
Schematic representation of the most likely routes to neural invasion by SARS-CoV-2. Left: orange depicts the olfactory neuroepithelium route; blue depicts the bloodstream route after lung invasion (reproduced from https://sq.wikipedia.org/wiki/Lemza). Right: red circles indicate brain areas with the highest SARS-CoV titers (figure kindly provided by the Servier Medical Art Department). All figures are reproduced under a CC BY-SA license.

It does not surprise that patients develop a variety of neurological symptoms (1). Indeed, SARS-CoV-2 is likely to infect the central nervous system early in the disease's course (2). Results from a large survey involving 2,343 European neurologists revealed headache (61.9%), myalgia (50.4%), anosmia (49.2%), ageusia (39.8%), impaired consciousness (29.3%), and psychomotor agitation (26.7%) as the most frequent neurological findings (3). In a recent experiment, viral replication within neurons soon after infection by SARS-CoV-2 in a human-induced pluripotent stem cell (iPSC)-derived BrainSphere model (4), supporting virus neurotropism. The neural consequences of viral infection were further tested in 47 patients with mild (*n* = 20), moderate (*n* = 9), or severe (*n* = 18) COVID-19, which were compared to 33 controls (5). Plasma levels of neurofilament light chain protein, a marker of neuronal injury, and glial fibrillary acidic protein, a marker of astrocyte damage, were higher in moderately or severely affected patients. Interestingly, astrocyte damage appeared to precede neuronal death. These findings suggest that brain injury may be more common than previously thought. COVID-19 should be viewed as a multisystemic disease, the involvement of the nervous system being noteworthy. Acute disseminated encephalomyelitis has been observed after SARS-CoV-2 infection in a patient without prominent clinical pulmonary symptoms (6). Some patients with COVID-19 have developed Guillain–Barré syndrome (7). These findings suggest that neuronal damage may concern not only the olfactory system and brainstem nuclei, as was initially suggested (8).

Olfactory loss may be the earliest neurological sign in COVID-19. One study conducted during the early pandemic in Italy showed that 13.5% of a small sample of patients with COVID-19 had developed hyposmia (9). A later study found olfactory dysfunction in 85.6% of 417 mild-to-moderate COVID-19 patients recruited from 12 European hospitals (10). A recent systematic review of 10 studies including 1,627 patients reported a 52.73% (95% CI, 29.64–75.23%) hyposmia prevalence in COVID-19 patients (11). Interestingly, hyposmia may precede other COVID-19 symptoms in a large number of cases (10), highlighting the earliness in brain tissue invasion and the relevance of its awareness.

The long-term consequences of coronavirus infections may be serious, as suggested by the observation that certain CoV strains are linked to neurodegenerative changes resulting in multiple sclerosis (1). The first observations date back to the 1980s. More recent studies have identified CoV-OC43, which shows serological cross-reactivity with SARS-CoV (12), more often in brain tissue of multiple sclerosis patients than that in control subjects (1). Both direct and indirect pathophysiological mechanisms have been proposed (13). Cross-reactivity between viral antigens and myelin may be a key mechanism (13).

Febrile or afebrile seizures, myelitis, meningitis, encephalitis, Guillain–Barré syndrome, and depression are among the manifestations observed upon IV infection. It is noteworthy that brain disease can develop even in the absence of respiratory symptoms (1). Seasonal IV infection can also lead to neurological complications. One study reported neurological alterations in 21 patients of a wide range of age, observing encephalitis as the most frequent clinical sign (1). Fifty percent of them showed neurological sequelae, sometimes including parkinsonism. Neuroinflammation after the activation of the microglia and other immune cells promotes neuronal death and protein aggregation (1), which may favor neurodegenerative diseases development in due course, as below discussed further.

Parkinson's disease (PD) affects nearly 6.1 million people globally. Our understanding of the pathophysiology of the disease has radically changed in recent decades. We now believe that PD is an umbrella disorder encompassing many genetic–molecular entities affecting many systems, resulting in a broad spectrum of motor and nonmotor features (14). The main histological finding is the presence of intracellular Lewy bodies composed of misfolded α-synuclein protein aggregates (14). Neuroinflammation, apoptosis, mitochondrial dysfunction, altered calcium homeostasis, inadequate protein degradation, and synaptic pathobiology have been cited as mechanisms resulting in either cell death and α-synuclein deposition or both (14).

Infections may play a role in PD development. A recent meta-analysis has shown that individuals with ongoing infections had a 20% higher PD risk compared with controls (15). Interestingly, IV infection was identified as one event that increased the risk of PD (15).

According to a recent theory, PD onset may be triggered by exposure to air pollutants, pesticides, heavy metals, head trauma, gastrointestinal microbiota perturbations, and pathogens (16) like the airborne viruses already discussed. The correlation between the routes of viral brain invasion and the findings of Braak and colleagues further supports this hypothesis. According to these authors, Lewy bodies can be first found at the brainstem and the olfactory cortex, long before damage to the substantia nigra results in the typical motor symptoms (17). As discussed earlier, viruses may reach the brainstem via a transsynaptic route and the olfactory cortex via the olfactory neuroepithelium (1, 2). While PD development requires not only triggers but also facilitators and aggravators (16), these pieces of evidence reinforce the potential connection with an airborne viral infection. So far, a potential triggering effect of CoV infection has not been reported. Notwithstanding, the above-discussed data support this idea.

What way viral infection may lead to developing a neurodegenerative disease is unclear. A local immune response leading to neuroinflammation is a likely candidate (13). Recent data show that α-synuclein may participate in the immune response, and infections may induce its upregulation (16). In turn, this molecule may activate microglia (18). Inflammatory cytokines and chemokines produced by microglia cells would amplify the inflammatory response (19), leading to neuronal death (20). In addition, neuroimmune responses to infection may lead to glutamate excitotoxicity (13), linked to neuronal degeneration (14).

The extent to which the novel SARS-CoV-2 respiratory virus pandemic is implicated in PD development should not be overlooked. This novel virus may infect millions of people, many likely being ever unaware. As said, central nervous system infection may occur in the absence of other symptoms (2). Noteworthy, smell alteration is being retrospectively recalled as an early symptom prodromal to later respiratory distress by infected people developing COVID-19 (9). In these cases, even if the immune system can control the infection and prevent an overt disease, the triggering of PD may have already taken place. Here again, smell alteration is early recalled by PD patients, as manifesting even years before PD diagnosis (14). The triggering effects may escalate upon repetitive exposure to the virus over the lifespan. Even if such effects are mild or moderate, the number of people exposed, reaching several million, suggests that the implications on PD should not be overlooked.

In sum, infection with CoV or IV respiratory viruses may increase the risk of developing PD over a lifetime. Pandemics of respiratory viruses appear a hallmark of the modern era and may be expected to reappear over time, according to experts. Besides the death toll, these pandemics may contribute to an increased worldwide burden of PD, which may only become noticeable many decades after the outbreaks. Health systems should be ready to tackle an eventual increase in PD burden. Notwithstanding, the infected population at risk for developing PD is an interesting target for testing disease-modifying or neuroprotective treatments.

## Author Contributions

All authors contributed to the article and approved the submitted version.

## Conflict of Interest

The authors declare that the research was conducted in the absence of any commercial or financial relationships that could be construed as a potential conflict of interest.
